# Genetic Polymorphisms of *IL-17F* and *TRAF3IP2* Could Be Predictive Factors of the Long-Term Effect of Infliximab against Crohn's Disease

**DOI:** 10.1155/2015/416838

**Published:** 2015-10-19

**Authors:** Shigetoshi Urabe, Hajime Isomoto, Tetsuya Ishida, Kazumi Maeda, Tatsuo Inamine, Shinji Kondo, Norihide Higuchi, Kayoko Sato, Ryohei Uehara, Hiroyuki Yajima, Haruhisa Machida, Chun Chuan Chen, Yasuhiro Fukuda, Fuminao Takeshima, Kazuhiko Nakao, Kazuhiro Tsukamoto

**Affiliations:** ^1^Department of Gastroenterology and Hepatology, Nagasaki University Hospital, 1-7-1 Sakamoto, Nagasaki 852-8501, Japan; ^2^Department of Gastroenterology, Oita Red Cross Hospital, 3-2-27 Chiyo-machi, Oita 870-0033, Japan; ^3^Department of Pharmacotherapeutics, Nagasaki University Graduate School of Biomedical Sciences, 1-7-1 Sakamoto, Nagasaki 852-8501, Japan; ^4^Department of Hospital Pharmacy, Nagasaki University Hospital, 1-7-1 Sakamoto, Nagasaki 852-8501, Japan

## Abstract

*Background*. We aimed to identify certain genes related to response to infliximab (IFX) and biomarkers to predict the IFX effect for Japanese Crohn's disease (CD) patients by performing an association study of single nucleotide polymorphisms (SNPs) in candidate genes in the interleukin- (IL-) 17 signaling pathway with response to IFX after 1 year of treatment. *Methods*. A total of 103 patients were divided into two groups, responders and nonresponders. Twenty-eight tag SNPs in 5 genes were genotyped. The frequencies of alleles and genotypes of each SNP were compared between responders and nonresponders in three different inheritance models. A genetic test was performed using a combination of the associated SNPs as biomarkers. *Results*. Multivariate logistic regression analysis indicated that the four variable factors, concomitant use of immunomodulators, penetrating disease, a G/G genotype of rs766748 in *IL-17F*, and a C/C or C/A genotype of rs1883136 in *TRAF3IP2*, independently contributed to response to IFX after 1 year of treatment. Genetic test using the polymorphisms of these genes perfectly predicted the responder and nonresponder CD patients with both concomitant use of immunomodulators and penetrating disease. *Conclusion*. *IL17F* and *TRAF3IP2* are one of IFX-related genes, useful as biomarkers of IFX response, and may be target molecules for new therapeutic drugs.

## 1. Introduction

Crohn's disease (CD) is involved in idiopathic inflammatory bowel disease (IBD) and is mainly characterized by chronic granulomatous inflammatory changes in the gastrointestinal tract. Although the etiology of CD is unknown, it can be attributed to numerous environmental factors, genetic predisposition, and excessive immune and inflammatory responses [1, 2]. In most cases, CD develops at a young age and its symptoms, such as abdominal pain, diarrhea, and bloody stool, undergo cycles of remission and relapse, eventually resulting in the impairment of the quality of the life of CD patients [3].

Treatments of CD are selected on the basis of the present site of the lesions, the degree of inflammation, the presence or absence of complications, and the previous response to treatment. Among medical therapies, 5-amino salicylic acid is often used for patients with mild disease severity, whereas steroids and/or anti-TNF-*α* antibodies, such as infliximab (IFX) and adalimumab, are used for patients with moderate or severe disease severity [4].

IFX is a chimeric anti-TNF-*α* monoclonal antibody that consists of the variable region of the murine anti-TNF-*α* antibody and the constant region of human IgG1. IFX inhibits the action of TNF-*α* by neutralizing the biological activity of soluble TNF-*α*, by damaging cells on membrane-bound TNF-*α*, and by dissociating TNF-*α* from its receptor [5]. IFX is widely available for the treatment of CD since 1991, when its usefulness has been confirmed in clinical settings worldwide. In Japan, clinical trials of IFX were started in 1996. In the ACCENT I randomized clinical trial carried out in North America, Europe, and Israel, about 58% of patients responded within 2 weeks to a single infusion of 5 mg/kg IFX. However, thereafter only 39% of these responders, who received repeated infusions of IFX every 8 weeks, were still in remission after 54 weeks of treatment [6]. Therefore, identification of biomarkers to predict the long-term therapeutic effect of IFX is warranted.

Interleukin- (IL-) 17 is an inflammatory cytokine that is secreted from Th17 cells. Within the IL-17 families, there are six ligands (IL-17A to F) and five receptors (IL-17RA to RE). In particular, intestinal Paneth cells express IL-17A and colonic epithelial cells produce IL-17F [7, 8]. After IL-17A forms a homodimerization with itself or a heterodimerization with IL-17F, their complex binds to a dimerized receptor consisting of IL-17RA and IL-17RC and subsequently transmits signals to downstream pathways through traf3-interacting protein 2 (TRAF3IP2), which share intracellular signal transduction molecules, such as I-*κ*B and NF-*κ*B, with the TNF-*α* signaling pathway [8–10]. Moreover, upregulation of parallel signaling pathways, including HGF and MET, to bypass the inhibited EGFR signaling pathway is known as one of the resistance mechanisms to gefitinib for patients with lung adenocarcinoma [11]. Thus, we speculate that the same resistance mechanism may occur to the second loss of response to IFX after 1 year of treatment. Indeed, IL-17A is overexpressed in inflammatory lesions and in the blood of patients with CD, multiple sclerosis, or systemic lupus erythematosus [12–14]. Furthermore, a correlation between the therapeutic effect of IFX and a decrease in the expression of IL-17RA after IFX administration has been observed in patients with rheumatoid arthritis [15]. Thus, IL-17 and its intracellular signaling pathways play a pivotal role not only in the pathogenesis of immune diseases including CD, but also in the response to IFX treatment.

Here, to assess as putative genes related to response to IFX, we examined a candidate gene-based association study by selecting several target genes involved in the IL-17 signaling pathway and investigated whether polymorphisms of these target genes are associated with the therapeutic effect of IFX for Japanese CD patients. We further investigated whether such polymorphisms could be used as new genetic biomarkers to identify Japanese CD patients showing response to IFX after the long-term treatment of 1 year.

## 2. Subjects and Methods

### 2.1. Subjects

The present study consisted of 113 unrelated Japanese CD patients treated with IFX in Oita Red Cross Hospital or Nagasaki University Hospital from 2004 to 2011.

The study protocol was approved by the Ethics Committee dealing with Human Genome and Gene Analysis at Oita Red Cross Hospital as well as at Nagasaki University. Written informed consent was obtained from each patient.

The diagnosis of CD was made based on the endoscopic, radiological, histological, and clinical criteria established by both the World Health Organization Council for International Organizations of Medical Sciences and the International Organization for the Study of Inflammatory Bowel Disease [16, 17]. Patients with indeterminate colitis, multiple sclerosis, systemic lupus erythematosus, or any other diagnosed autoimmune diseases were excluded from this study.

### 2.2. Definition of the Therapeutic Effect of IFX

Since Crohn's disease activity index (CDAI) of more than 150 [18] is regarded as active-phase CD, responders to IFX were defined as those showing a decrease in CDAI of less than 150 and an improvement in clinical manifestations, laboratory data, and/or endoscopic findings. Nonresponders to IFX were defined as those showing no change in the CDAI value or exacerbation of disease activity.

### 2.3. Study Design

**Figure 1:**
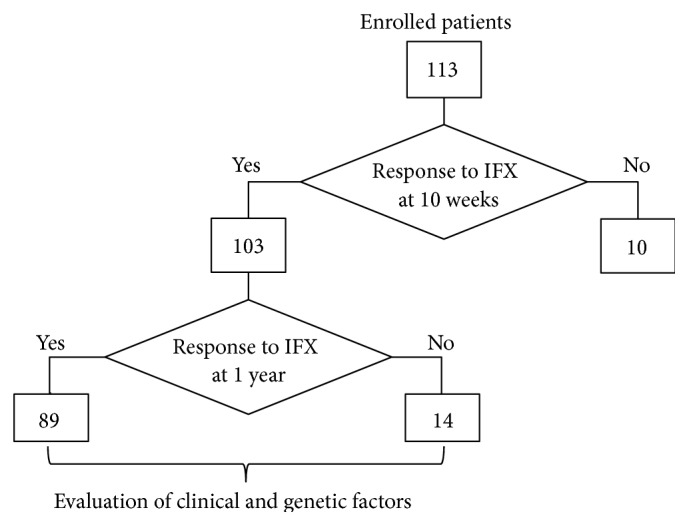
Flowchart of study design. A total of 113 CD patients were enrolled in this study. Of these patients, at the end of the 10-week IFX treatment, 103 patients showed response to IFX, 8 patients indicated loss of response to IFX, and IFX treatments of 2 patients were stopped due to side reactions of IFX. After 1 year of IFX treatment, 89 of the 103 patients still showed response to IFX, but the other 14 patients showed loss of response to IFX. CD: Crohn's disease; IFX: infliximab.

Of the 113 CD patients enrolled in this study, 103 patients who had shown response to IFX after 10 weeks of IFX treatment were subjected to this association study (Figure 1). These 103 responders to IFX at the end of the 10-week treatment were then divided into two groups, responders (*n* = 89) and nonresponders (*n* = 14), based on the presence or absence of IFX effect after the long-term IFX treatment of 1 year as shown in Figure 1. In addition, Table 1 shows the clinical characteristics including mean age, gender, smoking, concomitant use of immunomodulators, colonic location, and disease behavior, of both responders and nonresponders after 1 year of treatment (Table 1).

### 2.4. Selection of Tag Single Nucleotide Polymorphisms in Candidate Genes

All candidate genes selected in this study are involved in the IL-17 signaling pathway, including the genes encoding interleukin-17A (IL17A/*IL17A*; OMIM #603149); interleukin-17F (IL17F/*IL17F*; OMIM #606496); interleukin-17 receptor A (IL17RA/*IL17RA*; OMIM #605461); interleukin-17 receptor C (IL17RC/*IL17RC*; OMIM #610925); and traf3-interacting protein 2 (TRAF3IP2/*TRAF3IP2*; OMIM #1607043).

All of the information regarding single nucleotide polymorphisms (SNPs) in the candidate genes was obtained from Japanese data in Tokyo (Rel 24/phase II Nov. 08, on NCBI B36 assembly, dbSNP b126), which are available on the International HapMap website [19]. Candidate tag SNPs were selected from all SNPs in each chromosomal region including 2 kb upstream of the gene with priority for a minor allele frequency of more than 20% in the International HapMap data. Subsequently, genotyped tag SNPs among the candidate tag SNPs were determined based on linkage disequilibrium (LD) tagging using the Haploview 4.1 software program (*r* ^2^ > 0.8) [20].

**Figure 2:**
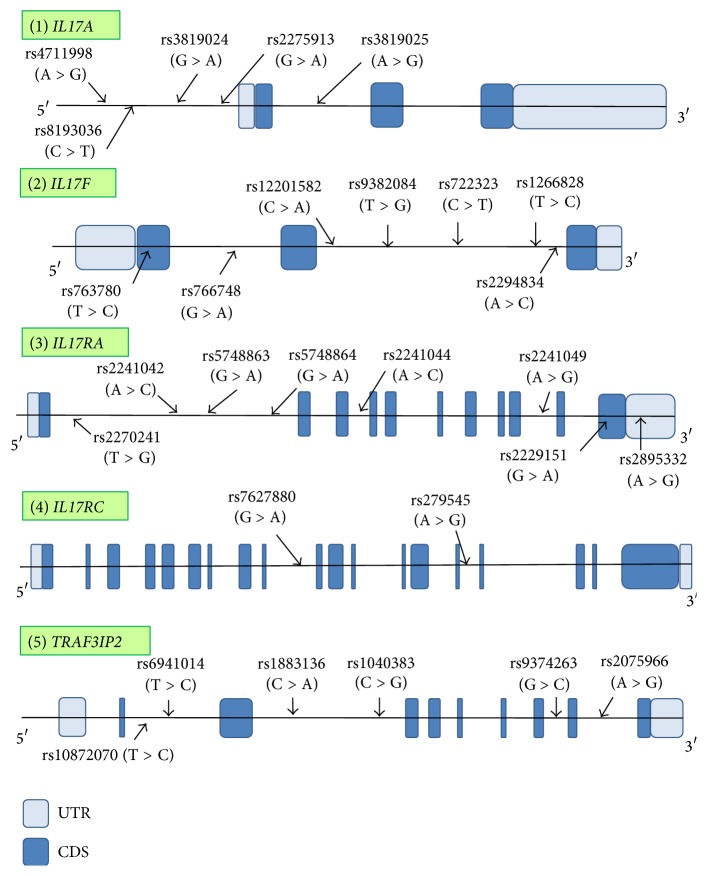
Gene structures and locations of genotyped tag SNP sites in each gene. The horizontal bars indicate the genomic sequences of candidate genes. Full boxes represent exons in each gene, and open boxes show the untranslated regions. The arrows indicate the positions of the genotyped tag SNP sites in this study and their names are presented above each site. SNPs: single nucleotide polymorphisms; CDS: coding sequence; UTR: untranslated region.

Twenty-eight tag SNPs, five in* IL17A*, seven in* IL17F*, eight in* IL17RA*, two in* IL17RC*, and six in* TRAF3IP2*, were selected as the genotyped tag SNPs. Information regarding the structure of the gene and the positions of the genotyped tag SNP sites in each candidate gene is shown in Figure 2.

### 2.5. Genotyping of Tag SNPs in Each Gene

Genomic DNA was extracted from whole blood samples using a DNA Extractor WB-Rapid Kit (Wako, Osaka, Japan) or a Quick Gene DNA Whole Blood Kit S (Fujifilm, Tokyo, Japan) with a Quick Gene-800 (Fujifilm) according to the manufacturer's protocol.

A total of 28 tag SNPs in 5 candidate genes were genotyped by polymerase chain reaction- (PCR-) restriction fragment length polymorphism (RFLP) or PCR-direct DNA sequencing method (Table 2) [21]. The polymorphic region was amplified by PCR with a GeneAmp PCR System 9700 thermal cycler (Life Technologies, Carlsbad, CA, USA) using 20 ng genomic DNA in a 25 *μ*L reaction mixture containing 1x GoTaq Green master mix (Promega, Madison, WI, USA) and 15 pmol each of forward and reverse primers (Table 2). The amplification protocol consisted of initial denaturation at 95°C for 2 minutes, followed by 30 or 35 cycles of denaturation at 95°C for 30 seconds, annealing for 30 seconds at the appropriate temperature for the primer pair (Table 2), extension at 72°C for 30 seconds, and final extension at 72°C for 5 minutes.

For the RFLP method, the PCR products were digested with the relevant restriction enzyme (Table 2), separated by electrophoresis on a 6% to 12% polyacrylamide gel (Nacalai Tesque, Kyoto, Japan) or a 2% ME-agarose gel (Nacalai Tesque), stained with ethidium bromide, and visualized using an ultraviolet transilluminator (Alpha Innotech Co., San Leandro, CA, USA).

For the direct DNA sequencing method, the PCR products were treated with ExoSAP-IT (Amersham Pharmacia Biotech, Piscataway, NJ, USA) and cycle sequenced using a BigDye Terminator v3.1 Cycle Sequencing FS Ready Reaction Kit (Life Technologies). The cycle sequencing was hot-started at 96°C for 30 seconds, followed by 25 cycles of denaturation at 96°C for 10 seconds, annealing at 50°C for 5 seconds, and extension at 60°C for 4 minutes using 1 pmol PCR forward or reverse primer. After the sequencing reaction solutions were purified using Sephadex G-50 superfine columns (Amersham Pharmacia Biotech), the samples were dried and sequenced with an ABI Prism 3100 Genetic Analyzer (Life Technologies).

### 2.6. Statistical Analyses

The mean age of IFX-responder CD patients and nonresponder CD patients after 1 year of treatment was presented as means ± standard deviation and was compared using the Mann-Whitney *U* test. The other clinical characteristics were compared using a chi-square or Fisher's exact test. The above statistical analyses were performed using the IBM SPSS Statistics 20 software package (IBM Japan, Tokyo, Japan) or Prism 6 (GraphPad Software, Inc., La Jolla, CA, USA).

The significance of deviation from the Hardy-Weinberg equilibrium (HWE) and LD between pairs of SNPs was analyzed using a chi-square test based on the expectation-maximization algorithm using the SNPAlyze 7.0 standard software package (Dynacom Inc., Chiba, Japan). The frequencies of alleles and genotypes were compared between responders and nonresponders after 1 year of treatment using the chi-squared or Fisher's exact test with odds ratio (OR) and 95% confidence interval in three different inheritance models: the allele, the minor allele dominant, and the minor allele recessive, using the SNPAlyze 7.0 standard software package.

In addition to univariate analyses, multivariate logistic regression analysis was carried out for analysis of the interaction of clinical environmental factors and putative genetic factors with the therapeutic effect of IFX after 1 year of treatment using JMP Pro 11 (SAS Institute Inc., Tokyo, Japan). A *P* value of less than 0.05 was considered to be statistically significant.

## 3. Results

### 3.1. Comparison of the Clinical Characteristics of Responders and Nonresponders to IFX

When the clinical characteristics of responders and nonresponders to IFX after 1 year of treatment were compared, there were significant differences in the concomitant use of immunomodulators as well as in the presence of penetrating disease behavior between the two groups (Table 1). Thus, the percentage of nonresponder CD patients concomitantly treated with immunomodulators was higher than that of the responders (57.1% versus 22.5%, *P* = 0.007). The percentage of nonresponder CD patients with penetrating disease was also higher than that of the responders (57.1% versus 20.2%, *P* = 0.006).

### 3.2. Association between Tag SNPs and Response to IFX after 1 Year of Treatment

Comparison of the distribution of alleles and genotypes of tag SNPs in each gene between responders and nonresponders to IFX after 1 year of treatment is shown in Table 3. The three tag SNPs, rs5748863 in* IL17RA* and rs10872070 and rs2075966 in* TRAF3IP2*, were excluded from the subsequent analyses because they were not in HWE.

Chi-square or Fisher's exact test in three different inheritance models indicated that the frequency of a heterozygous G/A genotype or a minor homozygous A/A genotype of rs766748 in* IL17F* in the minor allele dominant model was significantly decreased in responders as compared to that in nonresponders (*P* = 0.019, OR = 0.203; Table 3). This result implies that there was a ~4.9-fold loss of response to IFX in the nonresponders with these genotypes after 1 year of treatment as compared to the responders. Conversely, possessing a major homozygous G/G genotype of rs766748 in* IL17F* indicated that there was a ~4.9-fold response to IFX in the responders with this genotype as compared to the nonresponders.

Moreover, the frequency of a minor homozygous A/A genotype of rs1883136 in* TRAF3IP2* in the minor allele recessive model was significantly lower in responders in comparison to that in nonresponders (*P* = 0.041, OR = 0.213; Table 3), indicating that this genotype is associated with a ~4.7-fold loss of response to IFX. Conversely, possessing a major homozygous C/C genotype or a heterozygous C/A genotype of rs1883136 in* TRAF3IP2* indicated a ~4.7-fold increase in response to IFX after 1 year of treatment.

### 3.3. The Interaction of Genetic and Environmental Factors on Response to IFX after 1 Year of Treatment

Univariate analyses of the differences between responders and nonresponders indicated that the environmental factors of nonconcomitant use of immunomodulators or nonpenetrating disease and the genetic factors of the G/G genotype of rs766748 in* IL17F* or the C/C or C/A genotype of rs1883136 in* TRAF3IP2* showed response to IFX after 1 year of treatment. We then performed multivariate logistic regression analysis of the influence of the interaction of these factors on response to IFX after 1 year of treatment. This analysis revealed that these four variable factors independently contributed to response to IFX (*P* = 0.0162, OR = 5.281, *P* = 0.0073, OR = 6.529, *P* = 0.0213, OR = 5.123, and *P* = 0.0149, OR = 10.43, resp.; Table 4).

### 3.4. Verification of Genetic Test for Prediction of Response to IFX

In order to better predict the response to IFX for CD patients after 1 year of treatment, we carried out a genetic test using a combination of the two independent genetic factors (*IL17F* and* TRAF3IP2* genotypes) with or without the concomitant use of immunomodulators and/or penetrating disease (Tables 5 and 6). With regard to the patients concomitantly treated with immunomodulators, the prediction panel indicated that both the G/G genotype of rs766748 in* IL17F* and the C/C or C/A genotype of rs1883136 in* TRAF3IP2* were strongly associated with response to IFX in the CD patients with use of immunomodulators (*P* = 0.0019, OR = 37.92; Table 5). In this genetic test, the sensitivity, specificity, positive predictive value, and negative predictive value were estimated at 70.0%, 100%, 100%, and 57.1%, respectively.

Moreover, when the CD patients with both the concomitant use of immunomodulators and penetrating disease were considered, the above described polymorphisms showed a close association with response to IFX with the sensitivity, specificity, positive predictive value, and negative predictive value all estimated as 100% (Table 6).

On the other hand, there were no significant associations with response to IFX after 1 year of treatment in the patients with other combinations of with or without the concomitant use of immunomodulators and/or penetrating disease (Supplemental Tables 1–6 in Supplementary Material available online at http://dx.doi.org/10.1155/2015/416838).

## 4. Discussion

The present study is the first report to show that each of the genetic factors, polymorphisms of* IL-17F* and* TRAF3IP2*, and the clinical risk factors, concomitant use of immunomodulators and penetrating disease, independently contributed to the therapeutic effect of IFX after the long-term (1 year) IFX treatment of Japanese CD patients. The CD patients possessing the G/G genotype of rs766748 in* IL-17F* or the C/C or C/A genotype of rs1883136 in* TRAF3IP2*, or having nonuse of immunomodulators or nonpenetrating disease behavior, showed good response to IFX after 1 year of treatment. Conversely, the patients possessing the G/A or A/A genotype of rs766748 in* IL-17F* or the A/A genotype of rs1883136 in* TRAF3IP2*, or having the concomitant use of immunomodulators or penetrating disease behavior, displayed the secondary loss of response to IFX after 1 year of treatment although they had presented good response to IFX at the end of the 10-week IFX treatment.

TRAF3IP2 is an IL-17R adaptor protein that is also referred to as NF-*κ*B activator 1. After a heterodimer of IL-17A and IL-17F binds to their receptor, that is, comprised of IL-17RA and IL-17RC, the IL17-IL17R complex signals induce the activation of TRAF3IP2 and TNF receptor-associated factor 6. This signaling pathway eventually leads to the production of proinflammatory cytokines through NF-*κ*B [8–10, 22–24]. Moreover, TRAF3IP2 interacts with other TRAF proteins such as TRAF3 and TRAF6 that play multiple signaling roles in the IL-17 and TNF-*α* signaling pathways [8, 22, 25, 26]. For these reasons, it seems reasonable to speculate that the polymorphisms in* IL-17F* and* TRAF3IP2* identified in this study may affect these signaling pathways. Although we did not functionally analyze these SNPs, it is possible that in the intestines of nonresponder CD patients with genetic backgrounds of the G/A or A/A genotype of rs766748 in* IL-17F* or of the A/A genotype of rs1883136 in* TRAF3IP2* may affect the slight gain-of-function of both IL-17F and TRAF3IP2, thereby leading to a certain level of activation of both the IL-17 and TNF-*α* signaling pathways. It is known that (1) downstream signal transduction molecules, including I-*κ*B and NF-*κ*B, are shared by the IL-17 and TNF-*α* signaling pathways [8–10], (2) IL-17R knock-out mice are protected from inflammatory diseases including CD [27], and (3)* TRAF3IP2*-deficient mice are protected against dextran sodium sulfate-induced colitis, which presents with IBD-like manifestations [28]. Thus, elevated production of proinflammatory cytokines through the activation of both the IL-17 and TNF-*α* signaling pathways, which may occur in the IFX nonresponders, can lead to perpetuation of the chronic intestinal inflammatory process and might thereby result in the secondary loss of response to IFX after 1 year of treatment. Since these patients showed good response to IFX at the end of the 10-week treatment, IFX treatment can inhibit the weak activation of the IL-17 and TNF-*α* signaling pathways that might occur due to these polymorphisms of* IL-17F* and* TRAF3IP2* until the 10-week IFX treatment of these patients. However, the secondary loss of response to IFX, which occurs in these CD patients in the period from the end of the 10 weeks to the end of the 1 year of IFX treatment, may be due to a number of factors. Thus, during this period, the diminution of IFX-induced inhibition due to other environmental and genetic factors may lead to activation of the IL-17 and TNF-*α* signaling pathways, thereby resulting in the secondary loss of response to IFX. For example, various clinical risk factors, including smoking, enhancement of IFX clearance, new production of anti-IFX antibodies, a decrease in the blood concentration of IFX, and other unknown environmental factors and host genetic variations, can also contribute to the secondary loss of response to IFX [29–35]. In this study, among the clinical factors such as smoking, concomitant use of immunomodulators, colonic location, and disease behavior that we analyzed, two factors, the concomitant use of immunomodulators and penetrating disease, independently contributed to response to IFX after 1 year of treatment of the CD patient population.

With regard to this association, even if the effect of IL-17F is weaker than that of IL-17A, the polymorphisms of* IL-17F* were associated with the therapeutic effect of IFX after 1 year of treatment, but not* IL-17A*. Although we did not carry out protein analysis of IL-17, we guess the possibilities that (1) the percentage of homodimerization of IL-17F protein may be higher in the responder CD patients, but lower in the nonresponders, (2) the level of the gain-of-function of the IL-17 signaling pathway due to the G/A or A/A genotype of rs766748 in* IL-17F* may be accelerated in comparison to other polymorphisms of* IL-17F*, and (3) the activation of the IL-17 signaling pathway may be independent of the polymorphisms of IL-17A. Additional studies are needed to elucidate this mechanism.

On the other hand, the production of proinflammatory cytokines through the IL-17 and TNF-*α* signaling pathways may be downregulated in the CD patients possessing the G/G genotype of rs766748 in* IL-17F* and/or the C/C or C/A genotype of rs1883136 in* TRAF3IP2*. Therefore, IFX-induced inhibition of the TNF-*α* signals may keep stronger effect on IFX response than various risk factors, thereby resulting in a good response to IFX after 1 year of treatment of these patients.

With regard to other host genetic variations, the previously reported association studies of rheumatoid arthritis (RA) have shown that some SNPs in genes encoding proteins, such as p38 mitogen-activated protein kinase, AF4/FMR2 family, member 3, CD226, protein tyrosine phosphatase receptor type C, Fc gamma receptors IIA and IIIA, and tumor necrosis factor receptor superfamily member 1B, are associated with response to anti-TNF treatment [36–40]. It has been suggested that these SNPs are also involved in the therapeutic effect of IFX in CD patients. It is necessary to confirm that these RA-responsible SNPs do contribute to the effect of IFX in CD patients.

From the perspective of using genetic biomarkers to predict response to IFX for CD patients after 1 year of treatment, the present genetic test using the associated SNPs as biomarkers for the CD patients with the concomitant use of immunomodulators showed the highest specificity as well as the positive predictive value of 100% with significant differences. In addition, when this test was limited to the CD patients with both concomitant use of immunomodulators and penetrating disease, each of the sensitivity, specificity, positive predictive value, and negative predictive value was 100% with significant differences. These data suggest that, even though the CD patients possessing these polymorphisms of* IL-17F* and* TRAF3IP2* have clinical risk factors including the concomitant use of immunomodulators and penetrating disease, they should be treated with IFX because of the 100% sensitivity and 100% positive predictive value of the test. Conversely, the patients not possessing these polymorphisms of* IL-17F* and* TRAF3IP2* should not be treated with IFX based on the 100% test value for the specificity and negative predictive value. Therefore, the CD patients possessing these polymorphisms of* IL-17F* and* TRAF3IP2* together with both the concomitant use of immunomodulators and penetrating disease can perfectly indicate the responders and nonresponders to IFX after 1 year of IFX treatment.

## 5. Conclusion

This is the first report that* IL-17F* and* TRAF3IP2* are IFX-related genes in Japanese CD patients. The combination of the polymorphisms in these two genes is useful as a new biomarker to predict response to IFX after 1 year of treatment. Since the activation of the IL-17 signaling pathway may be associated with the secondary loss of response to IFX, the signal transduction molecules including IL-17F and TRAF3IP2 in the IL-17 signaling pathway might be possible new therapeutic targets for a part of CD and/or therapeutic agents to overcome the secondary loss of response to IFX treatment.

## Supplementary Material

Supplemental Table 1: Evaluation of a genetic test for response to IFX after 1 year of treatment of the CD patients without concomitant use of immunomodulators.
Supplemental Table 2: Evaluation of a genetic test for response to IFX after 1 year of treatment of the CD patients with penetrating disease.
Supplemental Table 3: Evaluation of a genetic test for response to IFX after 1 year of treatment of the CD patients without penetrating disease.
Supplemental Table 4: Evaluation of a genetic test for response to IFX after 1 year of treatment of the CD patients with concomitant use of immunomodulators plus non-penetrating disease.
Supplemental Table 5: Evaluation of a genetic test for response to IFX after 1 year of treatment of the CD patients with non-concomitant use of immunomodulators plus penetrating disease.
Supplemental Table 6: Evaluation of a genetic test for response to IFX after 1 year of treatment of the CD patients with non-concomitant use of immunomodulators plus non-penetrating disease

## Figures and Tables

**Table 1:** Comparison of the clinical characteristics of responders and nonresponders to IFX after 1 year of treatment of CD patients.

Characteristics	CD patients		*P* value
	Responders	Nonresponders	
Number	89	14	
Age, mean ± SD (years)	35.4 ± 12.9	37.8 ± 10.3	0.368
Male/female (%)	50/39 (56.2/43.8)	9/5 (64.3/35.7)	0.773
Smoking (%)	17 (19.1)	5 (35.7)	0.172
Concomitant use of immunomodulators (%)	20 (22.5)	8 (57.1)	0.007
Colonic location (%)^*∗*^			
Ilium	16 (18.0)	2 (14.3)	1.000
Colon	17 (19.1)	3 (21.4)	1.000
Ileocolon	56 (62.9)	9 (64.3)	1.000
Disease behavior (%)^*∗*^			
Nonstricturing/penetrating	43 (48.3)	5 (35.7)	0.409
Stricturing	37 (41.6)	3 (21.4)	0.238
Penetrating	18 (20.2)	8 (57.1)	0.006

^*∗*^Classification according to Montreal Classification for CD.

IFX: infliximab; CD: Crohn's disease; SD: standard deviation.

**Table 2:** Information regarding the genotyping of tag SNPs in candidate genes.

Gene	Tag SNP	Major > minor	Sequence of primers (5′ to 3′)		Annealing temperature (°C)	Cycle number	Analytical method (restriction enzyme)
			Forward	Reverse			
*IL17A*	rs4711998	A > G	TGTCCTCCAATTCCCTTTTG	GAGACATGATGGGGGAAAGA	60	30	PCR-RFLP (*Bpu* E I)
	rs8193036	C > T	TCTTTCCCCCATCATGTCTC	GTTCCAACCCTGCATGCTAC	60	30	PCR-direct DNA sequencing
	rs3819024	G > A	AGGCACAAACTCATCCATCC	GTCAGAACCCAGCGTTTCAT	60	30	PCR-direct DNA sequencing
	rs2275913	G > A	GTTTCCGGAATTGTCTCCAC	CCCAGGAGTCATCGTTGTTT	60	30	PCR-RFLP (*Bsl* I)
	rs3819025	A > G	AGTTTCCGGAATTGTCTCCA	CAGCTGCCAGAAGAGTTATGC	60	30	PCR-direct DNA sequencing

*IL17F*	rs763780	T > C	CACTGGTGCTCTGATGAGGA	CTGCATCAATGCTCAAGGAA	60	35	PCR-RFLP (*Hsp* 92 II)
	rs766748	G > A	GTGGGAATGAGTGGAGGAGA	TCCACTCGCTAAGCTGGACT	60	30	PCR-RFLP (*Afa* I)
	rs12201582	C > A	ACATTACGCAAAACCAACGA	TCATCCCCAGTAAGGGTCAG	60	30	PCR-direct DNA sequencing
	rs9382084	T > G	ATCAACTTTCATCCCCCACA	CGCCATAGCAGTTTGTCAAG	60	35	PCR-RFLP (*Alu* I)
	rs722323	C > T	GGATTTCTCTGAGAGGTGCTG	GGAAATCAGATGATGTCTGCAA	60	30	PCR-direct DNA sequencing
	rs1266828	T > C	CCCTGGATGGAAGAAATGAA	CATCAAAGCCTATGCCCCTA	60	30	PCR-RFLP (*Hpy* CH4 V)
	rs2294834	A > C	TTTGATTGGGGTCTTTTTGG	GGGATTACAGGCAACTGACC	60	30	PCR-direct DNA sequencing

*IL17RA*	rs2270241	T > G	CTGCGACTCCTGGACCAC	GTATTCCACACCGCAACTCC	60	35	PCR-direct DNA sequencing
	rs2241042	A > C	AGCTGCTTGCACAACTGCTA	CGCTTCTGGCATCTTTCTC	60	30	PCR-RFLP (*Hha* I)
	rs5748863	G > A	CAGGCGTGAGCCATGAAATT	GGCGCAGGGATCTACTGTTT	61	35	PCR-RFLP (*Bsa* A I)
	rs5748864	G > A	CTTGGTCTGGGTCTTCGTTG	GGAACCTCCACATGTTCCAC	60	30	PCR-RFLP (*Hpy* CH4 IV)
	rs2241044	A > C	TATGGGAACCAGAGCACCTC	GGTCCCAGGATGAAGAAGGT	60	35	PCR-RFLP (*Tsp* R I)
	rs2241049	A > G	ATGACCCTAGGCTGCTCCTT	GCGGGGGTTAACTCCTTAGT	60	30	PCR-RFLP (*Hpy* 188 I)
	rs2229151	G > A	TCATCTACTCAGCCGACCAC	GGAGCACAGGACGATGATCT	60	30	PCR-RFLP (*Hpy* 188 III)
	rs2895332	A > G	GCGTCCTTGAGGCTCCATTA	CTGGCCCATTCAGCGTTTAC	60	30	PCR-RFLP (*Hpy* CH4 IV)

*IL17RC*	rs7627880	G > A	CAAGGTCTCTTGTGCTTGC	GGATGCACTCATTCAGCAG	58	30	PCR-RFLP (*Mse* I)
	rs279545	A > G	CAGCCCTGGGAAAGTTAAG	CTGTCAAGATCCCCACTCC	60	30	PCR-RFLP (*Bse* Y I)

*TRAF3IP2*	rs10872070	T > C	CAAGCCTAGGCCATAAGCAG	TCCCACGTAGTCACCATTCA	60	30	PCR-RFLP (*Hsp* 92 II)
	rs6941014	T > C	AGCAGAGGGTGAGAGCATGT	TTGCTGATGAGCCTGAGATG	60	30	PCR-RFLP (*Hae* III)
	rs1883136	C > A	AATAGCTTCCCTGCGGACTT	GTGGGAGTTCCTGCAACAGT	60	30	PCR-RFLP (*Ssp* I)
	rs1040383	C > G	AGGCAACCAACTGGCAATAC	CTTTCTCCCAGGTTGCACAT	60	30	PCR-RFLP (*Xsp* I)
	rs9374263	G > C	TTTAACCAGGCCCACATGAT	TCAGGAGAGGAGCTGTTGGT	60	30	PCR-RFLP (*Hae* III)
	rs2075966	A > G	GAAATTGGCGATGGTATTGG	TCACACCTCCAGACATTTGC	60	30	PCR-RFLP (*Xsp* I)

SNP: single nucleotide polymorphism; 3′-UTR: 3′ untranslated region; PCR: polymerase chain reaction; RFLP: restriction fragment length polymorphism.

**Table 3:** Allele and genotype comparisons in three inheritance models between responders and nonresponders to IFX after 1 year of treatment of CD patients.

Gene symbol	Tag SNP (Major > minor)	Genotype	Number of genotypes (%)		Inheritance model^*∗*^	*P* value	OR	95% CI
			Responders	Nonresponders				
			*n* = 89	*n* = 14				
*IL17A*	rs4711998 A > G	MAF	0.337	0.321	Allele	0.870	1.073	0.458–2.517
		A/A	39 (43.8)	6 (42.9)				
		A/G	40 (44.9)	7 (50.0)	Dominant	0.946	0.962	0.308–3.002
		G/G	10 (11.2)	1 (7.1)	Recessive	1.000	1.646	0.194–13.96
	rs8193036 C > T	MAF	0.438	0.393	Allele	0.653	1.205	0.534–2.722
		C/C	32 (36.0)	5 (35.7)				
		C/T	36 (40.4)	7 (50.0)	Dominant	1.000	0.990	0.305–3.208
		T/T	21 (23.6)	2 (14.3)	Recessive	0.730	1.853	0.384–8.954
	rs3819024 G > A	MAF	0.421	0.423	Allele	0.669	0.840	0.377–1.870
		G/G	33 (37.1)	5 (35.7)				
		G/A	37 (41.6)	5 (35.7)	Dominant	1.000	0.943	0.291–3.053
		A/A	19 (21.3)	4 (28.6)	Recessive	0.509	0.679	0.191–2.406
	rs2275913 G > A	MAF	0.343	0.393	Allele	0.605	0.806	0.355–1.828
		G/G	40 (44.9)	5 (35.7)				
		G/A	37 (41.6)	7 (50.0)	Dominant	0.575	0.681	0.211–2.194
		A/A	12 (13.5)	2 (14.3)	Recessive	1.000	0.935	0.186–4.707
	rs3819025 G > A	MAF	0.292	0.321	Allele	0.752	0.871	0.370–2.052
		G/G	47 (52.8)	7 (50.0)				
		G/A	32 (36.0)	5 (35.7)	Dominant	0.845	0.894	0.289–2.760
		A/A	10 (11.2)	2 (14.3)	Recessive	0.666	0.759	0.148–3.897

*IL17F*	rs763780 T > C	MAF	0.157	0.071	Allele	0.385	2.427	0.545–10.81
		T/T	63 (70.8)	12 (85.7)				
		T/C	24 (27.0)	2 (14.3)	Dominant	0.341	2.476	0.518–11.85
		C/C	2 (2.2)	0 (0)	Recessive	1.000	0.829	0.038–18.17
	rs766748 G > A	MAF	0.247	0.429	Allele	0.045	0.438	0.192–0.997
		G/G	51 (57.3)	3 (21.4)				
		G/A	32 (36.0)	10 (71.4)	Dominant	0.019	0.203	0.053–0.779
		A/A	6 (6.7)	1 (7.1)	Recessive	1.000	0.940	0.105–8.454
	rs12201582 C > A	MAF	0.112	0.143	Allele	0.750	0.760	0.239–2.414
		C/C	70 (78.7)	10 (71.4)				
		C/A	18 (20.2)	4 (28.6)	Dominant	0.509	0.679	0.191–2.406
		A/A	1 (1.1)	0 (0)	Recessive	1.000	0.554	0.021–14.29
	rs9382084 T > G	MAF	0.433	0.357	Allele	0.453	1.372	0.600–3.141
		T/T	34 (38.2)	4 (28.6)				
		T/G	33 (37.1)	10 (71.4)	Dominant	0.564	0.647	0.188–2.227
		G/G	22 (24.7)	0 (0)	Recessive	0.037	9.671	0.554–168.8
	rs722323 C > T	MAF	0.438	0.429	Allele	0.924	1.040	0.465–2.326
		C/C	31 (34.8)	4 (28.6)				
		C/T	38 (42.7)	8 (57.1)	Dominant	0.768	0.748	0.217–2.584
		T/T	20 (22.5)	2 (14.3)	Recessive	0.729	1.739	0.359–8.425
	rs1266828 T > C	MAF	0.146	0.143	Allele	1.000	1.026	0.329–3.201
		T/T	66 (74.2)	10 (71.4)				
		T/C	20 (22.5)	4 (28.6)	Dominant	0.757	0.871	0.249–3.051
		C/C	3 (3.4)	0 (0)	Recessive	1.000	1.173	0.058–23.94
	rs2294834 A > C	MAF	0.157	0.179	Allele	0.783	0.859	0.301–2.449
		A/A	65 (73.0)	9 (64.3)				
		A/C	20 (22.5)	5 (35.7)	Dominant	0.530	0.665	0.202–2.184
		C/C	4 (4.5)	0 (0)	Recessive	1.000	1.526	0.078–29.90

*IL17RA*	rs2270241 T > G	MAF	0.365	0.429	Allele	0.519	0.767	0.342–1.721
		T/T	34 (38.2)	5 (35.7)				
		T/G	45 (50.6)	6 (42.9)	Dominant	1.000	0.899	0.278–2.908
		G/G	10 (11.2)	3 (21.4)	Recessive	0.379	0.464	0.110–1.952
	rs2241042 A > C	MAF	0.225	0.286	Allele	0.478	0.725	0.297–1.769
		A/A	52 (58.4)	6 (42.9)				
		A/C	34 (38.2)	8 (57.1)	Dominant	0.275	0.534	0.171–1.668
		C/C	3 (3.4)	0 (0)	Recessive	1.000	1.173	0.058–23.94
	rs5748864 G > A	MAF	0.478	0.536	Allele	0.567	0.792	0.356–1.761
		G/G	24 (27.0)	3 (21.4)				
		G/A	45 (50.6)	7 (50.0)	Dominant	0.757	0.739	0.190–2.878
		A/A	20 (22.5)	4 (28.6)	Recessive	0.734	0.725	0.205–2.560
	rs2241044 A > C	MAF	0.292	0.357	Allele	0.486	0.743	0.321–1.717
		A/A	43 (48.3)	5 (35.7)				
		A/C	40 (44.9)	8 (57.1)	Dominant	0.407	0.594	0.185–1.915
		C/C	6 (6.7)	1 (7.1)	Recessive	1.000	0.940	0.105–8.454
	rs2241049 A > G	MAF	0.326	0.429	Allele	0.286	0.644	0.286–1.451
		A/A	43 (48.3)	6 (42.9)				
		A/G	34 (38.2)	4 (28.6)	Dominant	0.704	0.802	0.257–2.502
		G/G	12 (13.5)	4 (28.6)	Recessive	0.225	0.390	0.105–1.444
	rs2229151 G > A	MAF	0.287	0.321	Allele	0.706	0.849	0.360–1.998
		G/G	44 (49.4)	7 (50.0)				
		G/A	39 (43.8)	5 (35.7)	Dominant	0.969	1.023	0.331–3.158
		A/A	6 (6.7)	2 (14.3)	Recessive	0.297	0.434	0.078–2.401
	rs2895332 A > G	MAF	0.348	0.286	Allele	0.517	1.336	0.556–3.209
		A/A	37 (41.6)	6 (42.9)				
		A/G	42 (47.2)	8 (57.1)	Dominant	0.928	1.054	0.337–3.295
		G/G	10 (11.2)	0 (0)	Recessive	0.350	3.830	0.212–69.09

*IL17RC*	rs7627880 G > A	MAF	0.112	0	Allele	0.082	7.372	0.433–125.5
		G/G	71 (79.8)	14 (100)				
		G/A	16 (18.0)	0 (0)	Dominant	0.122	7.503	0.427–131.8
		A/A	2 (2.2)	0 (0)	Recessive	1.000	0.829	0.038–18.17
	rs279545 A > G	MAF	0.213	0.179	Allele	0.806	1.249	0.445–3.503
		A/A	55 (61.8)	9 (64.3)				
		A/G	30 (33.7)	5 (35.7)	Dominant	1.000	1.113	0.344–3.600
		G/G	4 (4.5)	0 (0)	Recessive	1.000	1.526	0.078–29.90

*TRAF3IP2*	rs6941014 T > C	MAF	0.365	0.500	Allele	0.173	0.575	0.258–1.282
		T/T	35 (39.3)	4 (28.6)				
		T/C	43 (48.3)	6 (42.9)	Dominant	0.560	0.617	0.179–2.123
		C/C	11 (12.4)	4 (28.6)	Recessive	0.120	0.353	0.094–1.321
	rs1883136 C > A	MAF	0.292	0.464	Allele	0.068	0.476	0.212–1.071
		C/C	44 (49.4)	5 (35.7)				
		C/A	38 (42.7)	5 (35.7)	Dominant	0.398	0.568	0.176–1.830
		A/A	7 (7.9)	4 (28.6)	Recessive	0.041	0.213	0.053–0.860
	rs1040383 C > G	MAF	0.528	0.357	Allele	0.257	1.609	0.703–3.679
		C/C	24 (27.0)	6 (42.9)				
		C/G	46 (51.7)	6 (42.9)	Dominant	0.224	2.031	0.638–6.465
		G/G	19 (21.3)	2 (14.3)	Recessive	0.729	1.629	0.335–7.914
	rs9374263 G > C	MAF	0.331	0.429	Allele	0.315	0.661	0.294–1.488
		G/G	40 (44.9)	6 (42.9)				
		G/C	39 (43.8)	4 (28.6)	Dominant	0.884	0.919	0.294–2.868
		C/C	10 (11.2)	4 (28.6)	Recessive	0.096	0.317	0.083–1.201

^*∗*^Allele: allele model; Dominant: the minor allele dominant model; Recessive: the minor allele recessive model.

IFX: infliximab; CD: Crohn's disease; SNP: single nucleotide polymorphism; MAF: minor allele frequency; OR: odds ratio; CI: confidence interval.

**Table 4:** The interaction of genetic and environmental factors for response to IFX after 1 year of treatment of CD patients.

Factor	OR (95% CI)	*P* value^*∗*^
Nonconcomitant use of immunomodulators	5.281 (1.360–23.09)	0.0162
Nonpenetrating	6.529 (1.651–30.41)	0.0073
G/G genotype of rs766748 in *IL17F*	5.123 (1.261–27.77)	0.0213
C/C or C/A genotype of rs1883136 in *TRAF3IP2*	10.43 (1.603–77.68)	0.0149

^*∗*^Factors were statistically analyzed by multivariate logistic regression analysis.

IFX: infliximab; CD: Crohn's disease; OR: odds ratio; CI: confidence interval.

**Table 5:** Evaluation of a genetic test for response to IFX after 1 year of treatment of the CD patients with concomitant use of immunomodulators.

Factor	Number of CD patients (%)		OR (95% CI)	*P* value^*∗*^
	Responders	Nonresponders		
	*n* = 20	*n* = 8		
Both G/G genotype of rs766748 in *IL17F* and C/C and C/A genotype of rs1883136 in *TRAF3IP2*	14 (70.0)	0 (0)	37.92 (1.890–761.1)	0.0019
Others	6 (30.0)	8 (100)		

^*∗*^Factors were statistically analyzed by Fisher's exact test.

IFX: infliximab; CD: Crohn's disease; OR: odds ratio; CI: confidence interval.

**Table 6:** Evaluation of a genetic test for response to IFX after 1 year of treatment of the CD patients with both concomitant use of immunomodulators and penetrating disease.

Factor	Number of CD patients (%)		OR (95% CI)	*P* value^*∗*^
	Responders	Nonresponders		
	*n* = 4	*n* = 5		
Both G/G genotype of rs766748 in *IL17F* and C/C and C/A genotype of rs1883136 in *TRAF3IP2*	4 (100)	0 (0)	99.00 (1.618–6059)	0.0079
Others	0 (0)	5 (100)		

^*∗*^Factors were statistically analyzed by Fisher's exact test.

IFX: infliximab; CD: Crohn's disease; OR: odds ratio; CI: confidence interval.
